# Sorting Nexin 27 Enables MTOC and Secretory Machinery Translocation to the Immune Synapse

**DOI:** 10.3389/fimmu.2021.814570

**Published:** 2022-01-12

**Authors:** Natalia González-Mancha, Cristina Rodríguez-Rodríguez, Andrés Alcover, Isabel Merida

**Affiliations:** ^1^ Department of Immunology and Oncology, Centro Nacional de Biotecnología-Consejo Superior de Investigaciones Científicas (CNB-CSIC), Madrid, Spain; ^2^ Institut Pasteur, Université de Paris, Unité Biologie Cellulaire des Lymphocytes, INSERM U1224, Ligue Nationale Contre le Cancer, Équipe Labellisée Ligue-2018, Paris, France

**Keywords:** T lymphocytes, polarization, immune response, centrosome, diacylglycerol kinase ζ, retromer, SNX27

## Abstract

Sorting nexin 27 (SNX27) association to the retromer complex mediates intracellular trafficking of cargoes containing PSD95/Dlg1/ZO-1 (PDZ)-binding C-terminal sequences from endosomes to the cell surface, preventing their lysosomal degradation. Antigen recognition by T lymphocyte leads to the formation of a highly organized structure named the immune synapse (IS), which ensures cell-cell communication and sustained T cell activation. At the neuronal synapse, SNX27 recycles PDZ-binding receptors and its defective expression is associated with synaptic dysfunction and cognitive impairment. In T lymphocytes, SNX27 was found localized at recycling endosomal compartments that polarized to the IS, suggesting a function in polarized traffic to this structure. Proteomic analysis of PDZ-SNX27 interactors during IS formation identify proteins with known functions in cytoskeletal reorganization and lipid regulation, such as diacylglycerol (DAG) kinase (DGK) ζ, as well as components of the retromer and WASH complex. In this study, we investigated the consequences of SNX27 deficiency in cytoskeletal reorganization during IS formation. Our analyses demonstrate that SNX27 controls the polarization towards the cell-cell interface of the PDZ-interacting cargoes DGKζ and the retromer subunit vacuolar protein sorting protein 26, among others. SNX27 silencing abolishes the formation of a DAG gradient at the IS and prevents re-localization of the dynactin complex component dynactin-1/p150^Glued^, two events that correlate with impaired microtubule organizing center translocation (MTOC). SNX27 silenced cells show marked alteration in cytoskeleton organization including a failure in the organization of the microtubule network and defects in actin clearance at the IS. Reduced SNX27 expression was also found to hinder the arrangement of signaling microclusters at the IS, as well as the polarization of the secretory machinery towards the antigen presenting cells. Our results broaden the knowledge of SNX27 function in T lymphocytes by showing a function in modulating IS organization through regulated trafficking of cargoes.

## Introduction

Precise regulation of intracellular transport and vesicle fusion is of great importance in polarized cells, which depend on the delivery of cargoes to specialized areas of the plasma membrane to carry out their functions (1–3). In T cells, intracellular trafficking plays an essential role in the establishment of the immune synapse (IS), allowing the transport of surface receptors, signaling, adhesion, and scaffold molecules towards the cell-cell contact site, as well as their removal from there (4–8). Moreover, this process favors the polarized secretion of cytokines, lytic proteins and additional cargoes towards the antigen presenting cell (APC), modulating cell-cell communication (9–13). In this context, the rapid repositioning of the microtubule-organizing center (MTOC) is functionally linked to polarized trafficking. Although the regulatory mechanisms driving MTOC polarization are not fully understood, the formation of a stable diacylglycerol (DAG) gradient represents the first step for MTOC translocation (14–17).

A relevant player in the regulation of polarized trafficking is the sorting nexin (SNX) 27-retromer complex. SNX27 belongs to the SNX family of proteins, which are involved in intracellular trafficking and endosomal signaling. SNX27 is unique, as it is the only member of its family containing a N-terminal PSD95/Dlg1/ZO-1 (PDZ) domain that allows interaction with proteins bearing a C-terminal class 1 PDZ-binding motif (PDZ-bm). In addition, it can simultaneously mediate interaction with the vacuolar protein sorting protein 26 (Vps26) subunit of the retromer complex, which increases cargoes binding affinity and thus favors their recycling (8, 18). Firstly discovered in yeast, the retromer is a protein complex consisting in the association of two subcomplexes: the cargo-selection subcomplex, composed by the trimer Vps26-Vps35-Vps29, and the membrane-deforming subcomplex, which senses and induces membrane tubulation and is formed by the SNX-BAR heterodimer Vps5-Vps17 (19, 20). In mammals, the retromer trimer is conserved and includes Vps26A/Vps26B, Vps35, and Vps29 proteins (21–23). SNX-BAR heterodimers are formed by association of SNX1/SNX2 with SNX5/SNX6 (24). The retromer regulates the endosome-to-*trans* Golgi transport and the endosome-to-plasma membrane recycling, preventing cargo degradation (25–27). This tubular-based endosomal traffic is favored by retromer interaction with cytoskeleton components such as the motor dynein/dynactin complex or the Wiskott-Aldrich syndrome protein and SCAR homologue (WASH) complex, which regulates actin polymerization (8, 28, 29).

Several studies reported that SNX27-retromer function as a mediator of retrograde trafficking that contributes to sustain cell polarization. In neurons, SNX27 localizes to recycling endosomes and traffics to spines, facilitating the synaptic delivery of receptors (30). In epithelial cells, SNX27 recruits the epithelial cell-cell junction protein zonula occludens (ZO)-1/2 to recycling endosomes and distributes it to tight junctions (31). Similarly, SNX27 in T lymphocytes locates to recycling endosomes and traffics to the IS. This depends on mechanisms that require the presence of an intact PDZ domain (32, 33). However, the contributions of SNX27 and its associated cargoes to the formation of the IS has not yet been explored.

Here, we investigated the contribution of SNX27 to the polarized trafficking of cargoes and to the establishment of the IS in Jurkat T cells. We described that SNX27 controls the traffic towards the cell-cell interface of reported PDZ-interactors, including the retromer subunit Vps26 and the DAG kinase (DGK) ζ. SNX27 silencing abolishes the formation of a DAG gradient at the IS, an effect probably related to defective retrograde transport of DAG-enriched membranes to the cell-cell interface. Impaired DAG accumulation at the IS results in deficient synaptic recruitment of the motor protein dynactin-1/p150^Glued^ and the PDZ-interactor centromere protein J (CENPJ). Moreover, the absence of a proper DAG accumulation correlates with an incorrect polarization of the MTOC, the inefficient reorganization of the microtubule network, and modest defects in actin clearance at the IS. Furthermore, we observed defects in the arrangement of signaling microclusters, as well as in the polarization of the secretory machinery towards the IS. Our results strongly support a role for the SNX27-retromer-complex in IS-directed transport of associated cargoes, highlighting a critical role of SNX27 in the correct polarization of the MTOC during antigen recognition in Jurkat T cells.

## Materials and Methods

### Reagents and Antibodies

Reagents used were: poly-L-lysine, bovine serum albumin (BSA), DAPI (all purchased from Sigma-Aldrich), RPMI-1640 and L-glutamine (Biowest), CMAC (CellTracker Blue 7-amino-4-chloromethylcoumarin), BODIPY 630/650, (both from Thermo Fisher), Prolong Gold (Invitrogen), and recombinant human intercellular adhesion molecule 1 (ICAM-1) (R&D systems). For antibody (Ab)-based stimulation, we used a mouse anti-human CD3 monoclonal Ab (300402, Biolegend). For immunofluorescence staining, we used anti-: β-tubulin (MAB3408, Merk Millipore), CENPJ (11517-1-AP, Proteintech), dynactin-1/p150^Glued^ (MA1-070 Thermo Fisher), SNX27 (ab77799, Abcam), pericentrin (ab4448, Abcam), phalloidin-AlexaFluor 488 (A12379, Thermo Fisher), phosphorylated CD3ζ (Y142) (558402, BD biosciences), phosphorylated ZAP70 (Tyr 319) (PA5-17815, Thermo Fisher), phosphorylated LAT (Tyr 220) (3584, Cell signaling), and Vps26 (AB181352, Abcam). The following fluorophore-conjugated secondary Ab were used: anti-rabbit IgG-AlexaFluor 488, anti-mouse IgG-AlexaFluor 488, anti-mouse IgG2a-AlexaFluor 647 (A11034, A-11029, A21241; Thermo Fisher), anti-rabbit IgG-Cy2, anti-rabbit IgG-Cy3, anti-rabbit IgG -Cy5, anti-mouse IgG1-Cy3, anti-mouse IgG-Cy3, anti-mouse IgG1-Cy5 (2338021, 111-166-046, 111-176-046, 115-165-205, 115-166-075, 115-175-166; Jackson ImmunoResearch), and anti-mouse IgG2b-FITC (2794539, Southern Biotech). For flow cytometry analysis, we used anti-mouse CD45-APC (17-0451-82, Thermo Fisher). For western blot, we used anti-SNX27 (ab77799, Abcam), anti-GAPDH (sc25778, Santa Cruz), anti-GFP (A11122, Invitrogen), anti-DGKζ (ab105195, Abcam), and anti-p150Glued (MA1-070 Thermo Fisher). For immunoprecipitation, GFP-Trap Agarose (gta-20, ChromoTek) was employed.

### Cell Lines, Culture, and Stimulation

Human leukemic Jurkat T cells were obtained from the American Type Culture Collection (TIB-152, clone E6-1). Triple parameter reporter cells (TPR) are a human Jurkat JE6.1-derived cell line transduced with NFAT-GFP, AP-1-mCherry and NF-κB-CFP generated as previously described (34).T cell stimulator (TCS) cells are Bw5147 cells (murine thymoma cell line) modified to stably express an anti-human CD3 single chain fragment anchored to the plasma membrane *via* a human CD14 stem (CD5L-OKT3scFv-CD14). A variant of TCS cells was further engineered by retroviral transduction to express high amount of human CD86 (TCS-CD86) (35). Both TPR and TCS were kindly donated by Dr. Peter Steinberger (Medical University of Vienna, Austria). Jurkat T, TPR and TCS cells were cultured in complete RPMI medium consisting on RPMI-1640 supplemented with 10% FBS and 2 mM L-glutamine.

To establish immune synapses, TCS, used as APCs, were labeled in complete RPMI medium containing 10 μM CMAC or 1 μM BODIPY 630/650 (1 h, 37°C, darkness). After being washed twice in PBS, TCS were added at 1:1 ratio on top of Jurkat T cells previously plated on poly-L-lysine-coated coverslips. Cells were incubated for the indicated times (37°C, 5% CO_2_), after which immunofluorescence was performed.

For signaling microclusters, actin and microtubules immunofluorescence, Jurkat T cells in complete RPMI medium (2 × 10^6^ cells/ml) were seeded onto poly-L-lysine-coated glass coverslips with plate-bound anti-human CD3 (2.5 µg/ml) and recombinant human ICAM-1 (1 µg/ml, 2 h, 37°C) for the depicted times.

### Plasmids and Transfection

Jurkat T cells in logarithmic growth phase (4-5 × 10^5^ cells/ml) were electroporated using the Gene Pulser II (Bio-Rad; 270 V, 975 µF) or the Neon Transfection System (ThermoFisher; 1200 V; 10 ms pulse width; 2 pulses). For transient SNX27 silencing, double-strand oligonucleotides encompassing the interfering sequence 5´-CCAGGUAAUUGCAUUUGAA-3´ (36) and a hairpin structure were cloned in the pSuper vector (Oligoengine) (32). pSUPER-shRNAi mouse DGKα (37) was used as a transfection control. For transient protein expression, 15 µg plasmid DNA were transfected. The pEGFP-C1-hSNX27 (WT/L67-77A/H114A) (18) were a kind gift from Dr. Peter Cullen (University of Bristol, UK). mCherry-DGKζ WT, pEFbos-GFP DGKζ-ΔETAV, pEGFP-C1b-PKCθ, and pEGFP-C1-CD63 were previously described (38–40). While silenced cells were harvested 72 h post-transfection, cells with transient protein expression were processed 24 h post-transfection.

### Analysis of Protein Expression by Western Blot and Immunoprecipitation

For western blot analysis, cell pellets were suspended in cold lysis buffer (10 mM HEPES pH 7.5, 15 mM KCl, 1 mM EDTA, 1 mM EGTA, 10% glycerol, and 1% Nonidet P-40) containing protease inhibitors (10 μg/ml each leupeptin and aprotinin, 1 mM phenylmethylsulfonyl fluoride, 1 mM sodium orthovanadate, 40 mM β-glycerophosphate, and 10 μM NaF), and incubated 15 min at 4°C. A constant amount per sample was run in sodium dodecyl sulfate polyacrylamide gel electrophoresis (SDS-PAGE). Fluorescent signal was visualized using the Odyssey CLx Imaging System (LI-COR). SNX27 silencing was validated for every experiment by western blot analysis of total cell lysates.

For protein-protein interaction analysis by immunoprecipitation, cells were lysed as described above and the protocol was performed according to manufacturer’s instructions (GFP-Trap Agarose, Chromotek). Immunoprecipitated proteins were analyzed by western blot.

### Dual-Luciferase IL-2 Reporter Assay

Jurkat cells were transfected with control or SNX27-targeting shRNAi constructs by electroporation. At 48 h post-transfection, cells were re-electroporated with 15 μg of an IL-2 promoter construct (Addgene) together with 200 ng of Renilla luciferase vector (Promega) as an internal control. After 24 h, cells were stimulated with TCS cells at a 2.5:1 ratio in 96-well plates for the indicated time points. Lastly, cells were harvested, lysed in passive lysis buffer (Promega; 20 min, 4°C) and assayed for luciferase activity using the Dual-luciferase Reporter Assay (Promega). Relative luciferase units (RLU) were calculated relative to Renilla luciferase values.

### Il-2 Detection in Culture Supernatants

Jurkat T cells transfected with control or SNX27-targeting shRNAi constructs were incubated with TCS cells at 2.5:1 ratio in a flat bottom 96 well plate in triplicates for the indicated time points. Then, ELISA test was performed on the culture supernatants according to manufacturer’s intructions (Human IL-2 ELISA MAX_TM_ Delux, Biolegend).

### NFAT, NFkB, and AP-1 Promoter Activity Assay

TPR cells transfected with shcontrol or shSNX27 were incubated with TCS at 2.5:1 ratio for the indicated time points. Then, cocultures were stained with anti-mouse CD45-APC in PBS staining buffer (PBS, 1% FBS, 0.5% BSA, 0.01% sodium azide) (30 min, 4°C, darkness) to exclude TCS cells from analysis. Expression of NFAT-GFP, NFκB-CFP and AP-1-Cherry was determined by flow cytometry using a CytoFLEX S Flow cytometer (Beckman Cloulter). Live cells were gated using forward and side scatter parameters. All conditions were carried out in triplicates and data were analyzed using FlowJo 10 software (FlowJo, Ashland, OR) and Prism 5.

### Immunostaining

For immunofluorescence labeling, cells were fixed with 2% PFA (15 min, RT). After washing twice with PBS, cells were blocked and permeabilized 30 min, RT (1% BSA, 0.1% triton in PBS). This buffer was also used throughout the procedure as staining buffer. Cells were incubated with primary antibodies (1 h, RT), PBS-washed, and incubated with the corresponding fluorophore-conjugated secondary Ab (30 min, RT). Coverslips were washed twice in PBS and mounted on glass slides using ProLong Gold. For microtubule and signaling microclusters staining, 2% PFA fixation was performed at 37°C and this step was followed by an ice-cold methanol fixation and permeabilization (20 min, RT) prior to Ab staining.

### Confocal Microscopy and Image Processing

Confocal images were acquired using: Leica TCS SP8 confocal microscope equipped with a Plan-Apochromat HCX PL APO 63 × 1.4 NA oil immersion objective, Zeiss Axiovert LSM 510-META inverted confocal microscope equipped with a Zeiss Plan-Apochromat 63 × 1.4 NA oil objective, Zeiss Axiovert LSM 700 inverted confocal microscope equipped with a Plan-Apochromat 63 × 1.4 NA oil objective, or Olympus Fluoview FV1000 confocal microscope equipped with a Plan-Apochromat 60 × 1.4 NA oil objective. Images were collected with FV10-ASW4.2 (Olympus), LAS X (Leica), or ZEN 2009 (Zeiss) acquisition softwares. Sets to be compared were acquired using the same acquisition settings.

For quantitative analysis of protein synaptic recruitment we employed two distinct methods of quantification. For membrane proteins, accumulation at the IS was compared with the other areas of the plasma membrane: in T cell/TCS conjugates, maximal intensity Z-projections of contiguous optical sections (0.2 µm-wide) that included all the three-dimensional fluorescence information were stacked. Analysis was carried out using an in-house designed plugin for Fiji software developed by Carlos Oscar Sorzano and updated by Ana Cayuela (32). This plugin measures the mean fluorescence intensity (MFI) in circular regions of interest at the background, the IS, and the plasma membrane of the T cell outside the IS. Then the IS/plasma membrane MFI ratio was calculated as: (MFI IS - MFI background)/(MFI plasma membrane – MFI background). To quantify synaptic recruitment of proteins present at internal compartments/endosomes, fluorescence accumulated at the IS was compared with total cell fluorescence. Images were acquired as explained above and were analyzed as previously described (32). Briefly: MFI of background, whole cell, and IS regions were computed. Afterwards, the IS/cell MFI ratio was calculated as: (MFI IS - MFI background)/(MFI whole cell - MFI Background). Ratio values are represented as dot plots, where each dot depicts an individual cell.

To analyze the recruitment of signaling microclusters to the IS, we carried out a previously reported quantification (41). Initially, the area of segmented cells was measured. Among each cell, microclusters present at the cell-coverslip optical section were defined as signal intensity maxima employing the “Find Maxima” method. A value of noise tolerance was arbitrarily set at each experiment according to its background. Finally, the number of clusters per cell was divided by the cell area to obtain the density of microclusters at the IS.

Microtubule network organization patterns at the IS were categorized by three researchers by observation of unlabeled images into two classes: pattern 1 (P1), radial microtubules anchoring at the periphery of the IS; pattern 2 (P2), non-radial microtubules unable to reach the periphery of the IS. Maximum intensity projections of 4 consecutive sections (0.8 µm) at the T cell-coverslip contact were generated using the Fiji software (42) and Huygens Pro software (version 14.10) was used to perform image deconvolution.

To quantify formation of the filamentous actin (F-actin) ring and its fluorescence intensity at the IS, analysis was carried out on a 1-μm-thick section at the cell-coverslip interface.Cells from multiple microscopic fields were manually defined, and their F-actin MFI was computed. To determine the F-actin phenotype at the IS, pixel intensity plots from a line across the IS were generated. Patterns were categorized in three different phenotypes. Ring: centrally depleted actin and F-actin ring at the periphery; intermediate: uneven depletion of actin with intensity dropping at the center of the IS; accumulated: F-actin all across the IS.

For centrosomal F-actin quantification, we first defined the centrosomal area. In order to do that, we carried out a radial line scan of F-actin fluorescence intensity from the MTOC of resting Jurkat T cells. The drop in fluorescence intensity was used a threshold to define the radius of the centrosomal area. Based on our results, we defined the centrosomal F-actin area as a circumference of 1 µm of radius around the MTOC. Background subtraction (rolling ball 50 pixel) on the average z-projection of the three planes above and below the MTOC was performed. Finally, the mean intensity of F-actin fluorescence in the 2 µm-diameter circle centered at the MTOC was measured.

To measure the ability of the MTOC, CENPJ, or CD63^+^ secretory lysosomes/multivesicular bodies (MVB) to translocate to the IS, maximum intensity projections of the whole cells (0.2 µm intervals) were generated using the ImageJ. The geometric center of MTOC, CENPJ, and MVB (MTOC^C^, CENPJ^C^ and MVB^C^) as well as the IS region were determined. The polarity index was computed diving the distance between MTOC^C^, CENPJ^C^ or MVB^C^ to the IS (“a” distance) by the distance from the IS to the distal pole of the T cell (“b” distance). This allowed polarity indexes to be normalized by cell size and shape. We considered polarization to occur when the polarity index was <0.25. Polarity index values are represented in graphs as dot plots, where each dot represents an individual cell. To analyze the relative aligned position of the mass center of the MTOC and the center of the IS, a first axis was defined by drawing a straight line along the T cell-APC contact area, which was later divided in six regions of equal length. Then, a second axis was defined with two points: the mass center of the cell and the center of the immune synapse. Parallel lines to the second axis, intersecting at the six division points of the first axis were drawn, establishing three areas at each side of the second axis. Finally, MTOC position was categorized in symmetric, intermediate or asymmetric depending on which of those three areas (closest-to-furthest to the second axis) the MTOC was found.

The distance between the MTOC and the nucleus was measured in three dimensions using the Image 3D suite plugin on ImageJ. After applying a median 3D filter, nucleus and MTOC were automatically threshold in 3D (using Otsu and iterative thresholding, respectively). Then, the shorter distance between the edges of these two organelles was computed.

### Statistical Analysis

For co-localization analysis, Pearson’s correlation and Mander’s overlap coefficients were calculated using the JACoP plugin (Just Another Co-localization plugin) (43) of ImageJ software.

Statistical analysis was performed with GraphPad Prism 5 software and samples were assumed to fit normality. Details about the data presentation, the experimental replication, and the adequate statistical tests used are included in the individual figure legends. Briefly, unpaired student’s *t*-test was used to analyze differences between two conditions. Unless otherwise indicated, two-way ANOVA with Bonferroni post-test was applied for multiple comparisons. The level of statistical significance is represented by * *p <*0.05; ** *p <*0.01; *** *p <*0.001; **** *p <*0.0001. Data are shown as mean ± standard error of the mean (SEM) unless otherwise specified.

## Results

### SNX27 Facilitates Vps26 Retromer Protein Polarization to the IS

**Figure 1:**
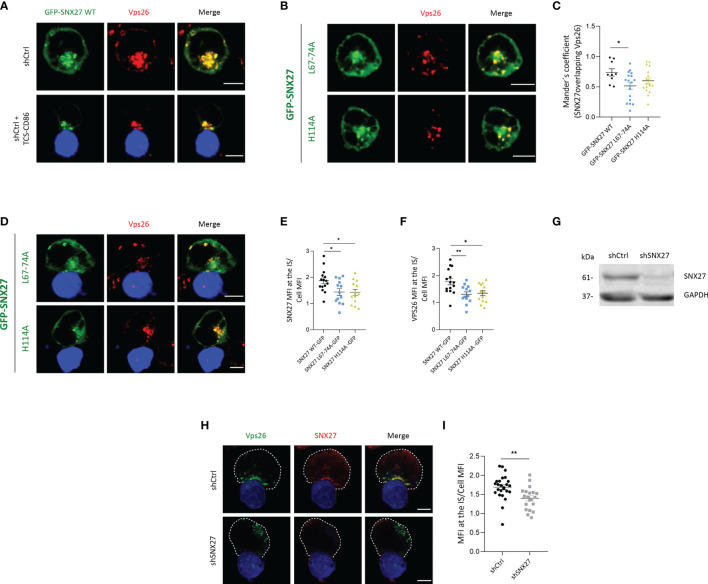
SNX27 interacts with Vps26 and contributes to its translocation to the IS. **(A, B, D)** Representative confocal images of Jurkat T cells transfected with plasmids encoding **(A)** GPF-SNX27 WT (green), **(B, D)** GFP-SNX27 L67-74A or GFP-SNX27 H114A (green) in basal conditions (**A**, top; **B**) or after incubation with TSC-CD86 (blue) (**A**, bottom; **D**). Cells were immunostained for Vps26. **(C)** Mander´s coefficient. Data in the graph are mean ± SEM of a representative experiment of two with similar results (GFP-SNX27 WT = 9 cells; GFP-SNX27 L67-744 = 18 cells; GFP-SNX27 H114A = 18 cells). **(E)** Quantification of SNX27 or **(F)** Vps26 translocation measured as the ratio of signal intensity at the IS compared with the total signal in the cell. Ratio values are displayed as dot plots, with each dot representing a single cell. Data are shown as mean ± SEM of an experiment (GFPSNX27 WT = 15 cells; GFP-SNX27 L67-744 = 13 cells; GFP-SNX27 H114A = 13 cells). **(G)** Western blot analysis of cell lysates from shControl and shSNX27 Jurkat T cells. **(H)** Representative maximum intensity projections from confocal images of control or SNX27-silenced Jurkat T cells incubated with TCS-CD86 (blue) and stained against Vps26 and SNX27. Dashed white line indicates cell contour. **(I)** Quantification of Vps26 translocation. Data are shown as mean ± SEM of a representative experiment of three with similar results (shCtrl = 25 cells; shSNX27 = 20 cells). Scale bars = 5 μm. Significance in **(C, E, F, I)** was determined by one-way ANOVA with Bonferroni correction (*p < 0.05; **p < 0.01).

The SNX27 PDZ domain engages proteins bearing a PDZ-bm and simultaneously associate the Vps26 subunit of the retromer complex (8, 18). We have previously shown that SNX27 mutants defective for either Vps26 binding (GFP-SNX27 L67-74A) or cargo interaction (GFP-SNX27 H114A) showed defective recruitment to the IS in experiments with Jurkat T cells and SEE-loaded Raji B cells (44). Although it constitutes a well stablished system to investigate IS organization upon encounter of an APC, the loading of Raji B cells with bacterial superantigens such as SEE may be heterogenous. In order to study the IS in a setting resembling physiology, we set Jurkat T cells to interact with TCS-CD86, a mouse thymoma cell line modified to express a membrane bound anti-CD3 antibody fragment and the CD86 costimulatory molecule (35). In agreement with their reported association (44), immunofluorescence analysis showed a strong colocalization between GFP-SNX27 WT and endogenous Vps26 in resting Jurkat T cells (**Figure 1A**, top). The interaction with TCS-CD86 resulted in a complete polarization of the SNX27/Vps26 positive compartment to the cell-cell contact area (**Figure 1A**, bottom). Analysis of the GFP-SNX27 L67-74A confirmed a marked reduction in the colocalization with Vps26 (**Figures 1B**, top; **1C**). A slight decrease in SNX27-Vps26 colocalization was also observed in cells expressing GFP-SNX27 H114A (**Figures 1B**, bottom; **1C**). As described (33), both mutants show defective polarization to the IS, but they affected differently the localization of endogenous Vps26 (**Figures 1D, E** **)**. The GFP-SNX27 L67-74A mutant showed partial accumulation at the IS, although this compartment was devoid of Vps26, that showed dispersal localization (**Figure 1D**, top). On the contrary, the mutant deficient in cargo binding retained colocalization with Vps26 in compartments that failed to polarize to the contact zone (**Figure 1D**, bottom). Deficient translocation of these SNX27 mutants correlated with a reduced accumulation of VPS26 at the IS (**Figure 1F**). These results suggest that the correct assembly of the SNX27-retromer complex together with its cargoes is required for its synaptic recruitment. This is further supported by a recent publication showing that impaired cargo recognition by SNX27 reduces retromer targeting to the plasma membrane (45).

The failure of Vps26 to relocate to the IS in GFP-SNX27 H114A overexpressing cells could be the result of sequestering the endogenous Vps26 away from its natural localization. To evaluate whether SNX27 was indeed required for Vps26 recruitment to the IS, we depleted cells for SNX27 **(** **Figure 1G** **)** and examined Vps26 localization upon engagement with TCS-CD86 cells. Analysis of endogenous proteins confirmed strong colocalization between SNX27 and Vps26 at the IS in control cells (**Figure 1H**, top). SNX27 silencing prevented the polarization of Vps26 positive vesicles, that in most cells appeared dispersed and opposite to the contact zone **(** **Figure 1H**, bottom; **1I**). These results confirm that PDZ-dependent interaction of SNX27 with Vps26 facilitates polarized traffic of this retromer subunit to the IS.

### SNX27 Regulates DAG Accumulation at the IS

**Figure 2:**
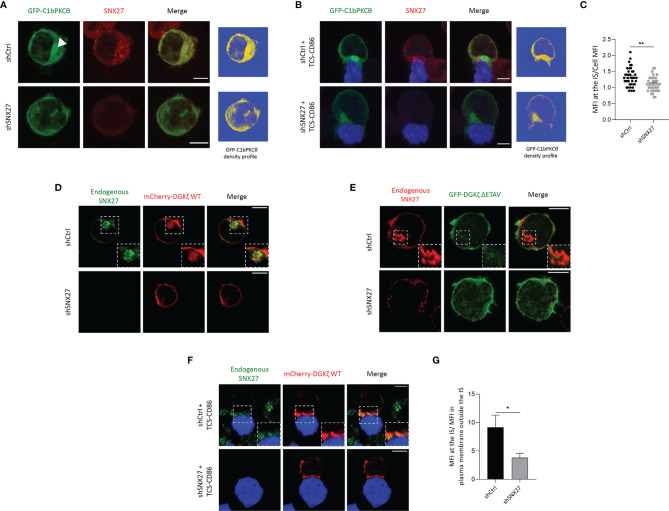
SNX27 controls DGKζ and DAG translocation to the IS. **(A, B)** Representative maximum intensity projections from confocal images of control or SNX27-silenced Jurkat T cells transfected with the C1 domain of PKCθ fused to GFP construct (GFP-C1bPKCθ, green) in basal conditions **(A)** or after incubation with TCS-CD86 (blue) **(B)**. Arrowhead points DAG in intracellular compartment. A single medial optical section from a representative experiment out of three is shown. Right panels represent the density profiles of DAG fluorescence obtained from the medial optical section. Color scale goes from blue (zero) to yellow (intermediate) to red (maximal). **(C)** Quantification of GFP-C1bPKCθ translocation measured as the ratio of signal intensity at the IS compared with total GFP-C1bPKCθ. Ratio values are displayed as dot plots, with each dot representing a conjugate. Data are shown as mean ± SEM of two independent experiments (shCtrl = 40 cells; shSNX27 = 35 cells). Significance determined by unpaired *t-test* (*p < 0.05; **p < 0.01). **(D–F)** Representative confocal images of control of SNX27-silenced Jurkat T cells transfected with **(D, F)** a plasmid encoding mCherry-DGKζ WT (red) or with **(E)** a GFP-DGKζ ΔETAV (green) construct in basal conditions **(D, E)** or after incubation with TCD-CD86 (blue) **(F)**. A representative experiment out of three in **(D)** or two in **(E)** is shown. Pearson´s correlation coefficient in shCtrl cells = 0.648 in (**D**). **(G)** Quantitative image analysis of mCherry-DGKζ WT accumulated at IS compared with mCherry-DGKζ WT located in plasma membrane regions outside the IS. Data are shown as mean ± SEM from a representative experiment of two with similar results Significance was determined by unpaired *t-test* (*p < 0.05). Scale bar = 5 μm.

Antigen recognition by T cell receptor (TCR) results in the rapid activation of retrograde traffic from the plasma membrane to the Golgi/recycling endosomes (RE), that orient to the contact area to facilitate a polarized traffic to the IS. Studies in Jurkat T cells and primary T lymphocytes have shown that the Golgi and RE are highly enriched in DAG (46). Upon T cell interaction with APCs, the rapid translocation of DAG-enriched organelles facilitates the trafficking of DAG-loaded vesicles to the IS, contributing to the accumulation of this lipid at the cell-cell junction (46). We investigated whether SNX27 silencing alters the polarization of DAG-positive compartments to the IS. As previously reported (46), Jurkat T cells overexpressing a fluorescent construct with high affinity for DAG (GFP-C1bPKCθ) presented intense fluorescence accumulation at internal compartments, which was easily visible in fluorescence density profiles **(** **Figure 2A**, top). SNX27 silencing abolished compact intracellular DAG staining, and the sensor appeared distributed throughout the plasma membrane **(** **Figure 2A**, bottom). DAG-positive compartments polarized to the cell-cell contact site upon TCS-CD86 engagement **(** **Figure 2B**, top). However, SNX27-silenced cells failed to accumulate DAG-positive organelles to this region**(** **Figure 2B**, bottom; **2C**). Previous studies from our group employing live cell imaging showed that upon cell-cell contact, a rapid burst of DAG at the IS is rapidly followed by polarization of DAG-enriched compartments to this region (46). The fast dynamics of DAG generation and traffic are difficult to capture in fixed images, as DAG is dispersed over the cell membrane after a short time after stimulation (39, 46). Nevertheless, we did observe a few control cells at initial contacts with TCS-CD86 displaying enrichment of DAG at the plasma membrane of the IS prior to the full translocation of SNX27 and DAG-enriched compartments **(** **Supplementary Figure 1** **).**

DGKζ transforms DAG into phosphatidic acid (PA), therefore contributing to the regulation of DAG content in T cells (46). DGKζ contains a PDZ-bm that provides high affinity interaction with SNX27 (47). Thus, we next explored the consequences of SNX27 silencing in the subcellular localization of its cargo DGKζ. In basal conditions, mCherry-DGKζ- was distributed between the plasma membrane and internal organelles that were positive for endogenous SNX27 **(** **Figure 2D**, top). Upon SNX27 silencing, this internal localization was lost and mCherry- DGKζ was mainly observed at the plasma membrane **(** **Figure 2D**, bottom). GFP-DGKζ ΔETAV, a DGKζ mutant lacking the last four amino acids of its PDZ-bm, showed a localization mainly restricted to the plasma membrane both in the presence and in the absence of SNX27 **(** **Figure 2E** **).** These data confirm the PDZ-dependent engagement of DGKζ with SNX27 and demonstrate that this interaction is indispensable for retrograde traffic of DGKζ to the Golgi/RE in basal conditions. Upon incubation with TCS-CD86 cells, control Jurkat T cells displayed strong accumulation of mCherry-DGKζ- at the IS **(** **Figure 2F**, top). Nevertheless, DGKζ accumulation at the cell-cell interface was impaired in SNX27-silenced cells and this DGK isoform remained randomly distributed at the plasma membrane **(** **Figure 2F**, bottom; **2G**). These data suggest that retrograde traffic of DGKζ to internal compartments is indispensable for this lipid kinase to reach the IS and support a non-previously reported role for SNX27 in the spatial localization of DGKζ, which could affect its functions.

### SNX27 Regulates MTOC Translocation to the IS

**Figure 3:**
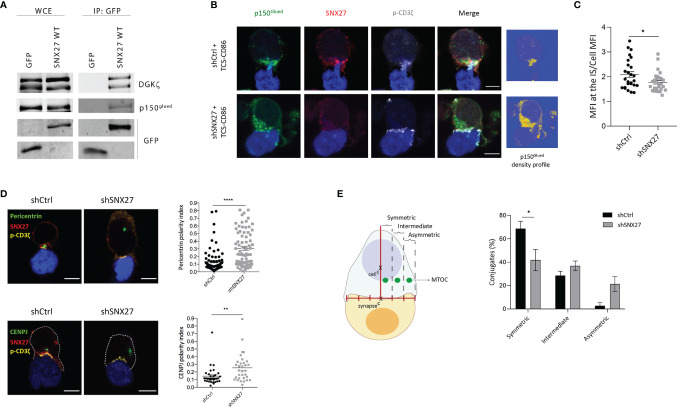
SNX27 is necessary for MTOC translocation and its symmetry with the center of the IS in Jurkat T cells. **(A)** Western blot analysis of GFP immunoprecipitates confirmed interaction of p150^glued^ with GFP-SNX27 WT. DGKζ was used as a positive control. **(B)** Representative maximum intensity projection from confocal z-stacks of control and SNX27-silenced Jurkat T cells incubated with TCS-CD86 (blue). Cells were stained for the indicated proteins: dynactin-1/p150^glued^ (green), SNX27 (red) and phosphorylated CD3ζ (gray)Density profiles of dynactin-1 fluorescence obtained from the maximum intensity projections are shown in (**B**, right). Color scale goes from blue (zero) to yellow (intermediate) to red (maximal). **(C)** Quantification of p150^glued^ translocation measured as the ratio of signal intensity at the IS compared with total p150^glued^. Ratio values are displayed as dot plots, with each dot representing a single cell. Data are shown as mean ± SEM of a representative experiment out of two run in duplicates (shCtrl = 26 cells; shSNX27 = 29 cells). Significance was determined by unpaired *t-test* (*p < 0.05; **p < 0.01, ****p < 0.0001). **(D)**, left] Representative confocal images of control and SNX27-silenced Jurkat T cells incubated with TCS-CD86 (blue). Cells were immunostained for pericentrin (MTOC, green) or CENPJ (green), SNX27 (red) and phosphorylated CD3ζ (yellow). Scale bar = 5 μm. [**(D)**, right] MTOC or CENPJ polarity index quantification calculated as the ratio between the distance from the MTOC or CENPJ to the IS and the distance from the IS to the distal pole of the cell. Polarity index values are displayed as dot plots, with each dot representing an individual cell. [**(E)**, left] Scheme depicting the strategy used to define MTOC symmetry (see methods section for detailed explanation). [**(E)**, right] Quantification of MTOC symmetry. Data in [**(D)**, right top; **(E)**] are mean ± SEM of three independent experiments in duplicates with shCtrl = 78 cells and shSNX27 = 74 cells, and in [**(D)**, left bottom] are mean ± SEM of a representative experiment out of three run in duplicates with shCtrl = 33 cells and shSNX27 = 32 cells. Significance was determined by unpaired *t* test in **(D)** and by two-way ANOVA with Bonferroni correction in **(E)**. (*p < 0.05; **p < 0.01; ****p < 0.0001).

The formation of a stable DAG gradient upon T cell-APC engagement is required for the rapid docking of the MTOC at the IS in a process regulated by the dynein-dynactin motors (14–16). Noteworthy, the proteomic study in IS-forming Jurkat T cells revealed the presence of the p150^Glued^ dynactin complex subunit among the SNX27 interactors (44). Immunoprecipitation studies validated the reported association between p150^Glued^ and SNX27 (**Figure 3A**). Therefore, we explored the consequences of SNX27 silencing on p150^Glued^ dynamics upon T cell activation. We confirmed p150^Glued^ colocalization with SNX27 at the IS, and observed that its synaptic recruitment was lost when SNX27 was depleted **(** **Figures 3B, C** **).** Phosphorylated ζ-chain was stained as a marker for correct IS formation to discard the possibility of an ineffective T cell-APC engagement. These results corroborated the proteomic data and demonstrated that SNX27 contributes to the dynamic recruitment of p150^Glued^ to the IS upon APC engagement.

The observed failure of SNX27-silenced T cells to accumulate DAG and defective p150^Glued^ recruitment at the synaptic region strongly suggests a role for SNX27 in MTOC translocation. Immunofluorescence assessment of conjugates established between Jurkat T cells and TCS-CD86 demonstrated that pericentrin (MTOC protein) polarity index to the IS was statistically higher when SNX27 was depleted **(** **Figure 3D**, top). Our proteomic analysis in IS-forming Jurkat T cells had previously identified CENPJ as a PDZ-dependent cargo of SNX27 (44). CENPJ is a highly conserved centrosomal protein essential for centrosome biogenesis (48). In agreement with the defective pericentrin polarization, SNX27-silenced Jurkat T cells displayed impaired CENPJ reorientation upon engagement with TCS-CD86 stimulatory cells **(** **Figure 3D**, bottom).

Disrupted ability of T cells to sustain DAG at the IS prevents MTOC translocation, limiting the correct alignment of the protein trafficking machinery. We analyzed the impact of SNX27 silencing on the relative aligned position of the mass center of the MTOC and the center of the IS **(** **Figure 3E**, left). Distribution was categorized into symmetric, intermediate or asymmetric. SNX27 silencing led to a reduced percentage of cells with a symmetric MTOC position **(** **Figure 3E**, right). These results indicate that SNX27 facilitates the PDZ dependent transport of centrosomal proteins to the central region of the cell-cell contact area.

### SNX27 Contributes to Peripheral, But Not Centrosomal, F-Actin Reorganization During T Cell Activation

**Figure 4:**
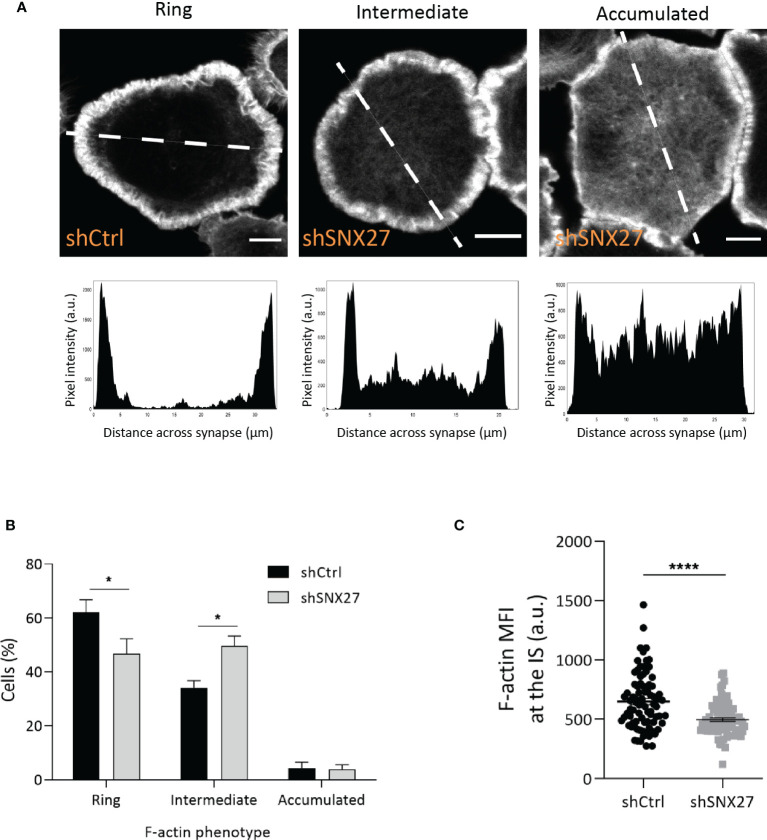
SNX27 silencing in Jurkat T cells alters F-actin remodeling at the IS. Control and SNX27-silenced Jurkat T cells were stimulated on anti-CD3 and ICAM-1-coated coverslips and immunostained for phalloidin-A488 (F-actin). **(A)** Representative confocal section at the cell-coverslip interface. F-actin organization patterns were classified in three phenotypes based on the pixel intensity plots across the synapse (lower panels): ring: F-actin ring at the periphery with depletion of F-actin at the center; intermediate: uneven depletion of F-actin at the center of the synapse, low F-actin ring or clearance; accumulated: F-actin across the synapse. White line in the pictures represents the distance across the synapse plotted in graphs. Analyses were performed on a 1-μm-thick section at the cell-coverslips contact. Scale bar = 5 μm. **(B)** Quantification of F-actin phenotype at the IS. Data are shown as mean ± SEM of three experiments in duplicates (shCtrl= 306; shSNX27 = 330). Significance was determined by two-way ANOVA with Bonferroni correction (*p < 0.05). **(C)** Quantification of F-actin MFI carried out on control and SNX27-silenced cells on a 1-μm-thick section at the cell-coverslips contact, regardless of their F-actin pattern. Data are shown as mean ± SEM of a representative experiment out of three run in duplicates (shCtrl=82; shSNX27 = 83). Significance was determined by unpaired *t -*test (*p < 0.05; ****p < 0.0001).

F-actin depolymerization at the center of the IS has been described to play an important function in centrosome polarization in T cells (49–53). Proteomic analysis in IS-forming T cells revealed a PDZ-independent interaction of SNX27 with the actin nucleators WASH complex, and Arp2/3 subunit ARPC5L (44). Using confocal microscopy, we evaluated the remodeling of F-actin at the IS in control and SNX27-silenced Jurkat T cells spread on anti-CD3 and ICAM-1-coated surfaces **(** **Figure 4A** **).** The establishment of pseudo-synapses on coverslips coated with stimulatory molecules facilitates the detailed analysis of molecule organization at the contact plane, and has been widely employed. F-actin organization patterns were classified in three phenotypes based on the F-actin pixel intensity plots across the synapse **(** **Figure 4A** lower panels): Ring, F-actin ring at the periphery with depletion of actin at the center; intermediate, uneven depletion of F-actin at the center of the synapse; accumulated, F-actin across the synapse. Although an F-actin ring was formed in both control and SNX27-silenced cells, we observed a modest defect in F-actin depletion across the center of the IS in SNX27-silenced cells, with a higher percentage of cells displaying an intermediate phenotype **(** **Figure 4B** **).** Besides, a significant reduction in F-actin MFI at the contact site was observed in the SNX27-silenced cells **(** **Figure 4C** **).** This suggests that failure in MTOC translocation could be prompted by a mild defect in F-actin depolymerization across the IS.

F-actin polymerization is also important around the centrosome to facilitate MTOC tethering to the nucleus in basal conditions (54–56). Studies in B cells have shown that F-actin nucleation around the centrosome is mediated by the nucleation-promoting factor WASH in combination with the Arp2/3 complex. Upon B lymphocyte activation, Arp2/3 translocates to the IS. This leads to F-actin depletion at the centrosomal area, favoring MTOC detachment and polarization to the cell-cell interface (54). The WASH complex associates to SNX27, so we wondered whether SNX27 deficiency would prevent centrosomal F-actin denucleation and MTOC detachment from the nucleus.

**Figure 5:**
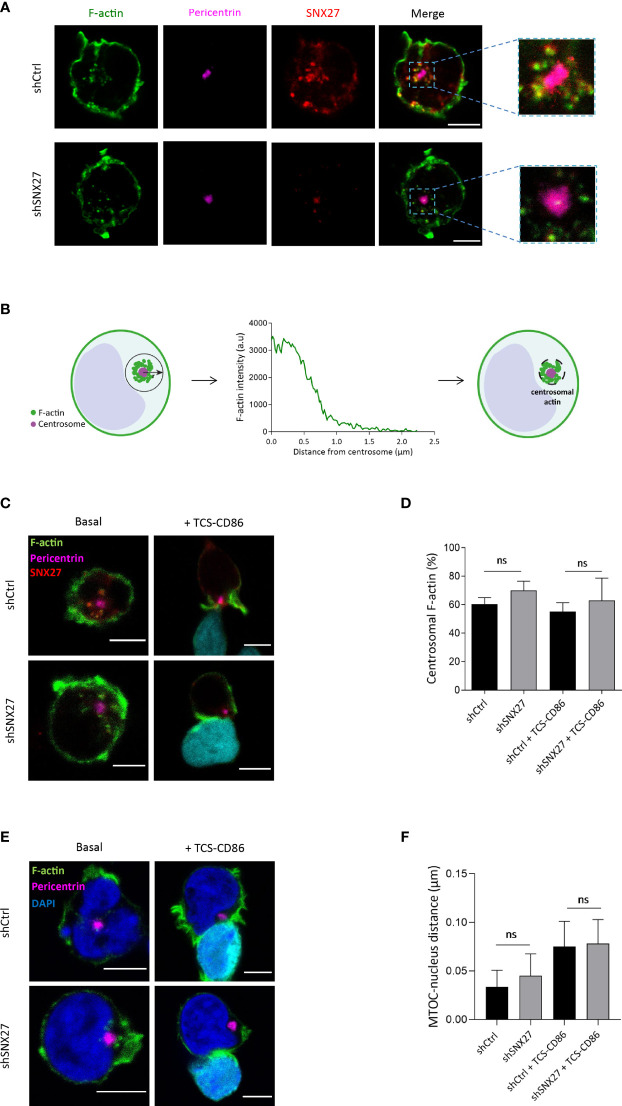
Centrosomal F-actin nucleation and MTOC detachment from the nucleus in Jurkat T cells is not SNX27-dependent. **(A, C, E)** Representative confocal images of control and SNX27-silenced Jurkat T cells in basal conditions [**(A, C)**, left] or incubated with TCS-CD86 [**(C, E)**, right] labeled with CMAC in **(C)** and Bodipy in **(E)**. Cells were stained for the indicated proteins: phallo-A488 (F-actin, green), SNX27 (red), pericentrin (MTOC, pink) or DAPI (blue) to label the nuclei. Medial optical sections from a representative experiment out of three **(A)** or two **(C, E)** are shown. **(B)** Scheme depicting the strategy used to define the centrosome-associated F-actin region. **(D)** Quantification of the percentage of centrosomal F-actin from cells shown in **(C)**. Values correspond to the fraction of F-actin fluorescence in a 1 μm-wide area around the centrosome relative to the total fluorescence in the cell. **(F)** 3D Quantification of the shorter distance between the edge of the MTOC and the border of the nucleus from cells shown in **(E)**. Significance was determined by one-way ANOVA with Bonferroni correction (ns, not significant). Scales bars = 5 μm.

As reported by Farina et al. (55), F-actin filaments were found in the cortical region of Jurkat T cells, as well as in the vicinity of centrosomes **(** **Figure 5A** **).** Centrosomal F-actin and SNX27 exhibited a similar distribution. To determine the centrosomal F-actin area, we followed a procedure previously described by Obino et al. (56). Briefly, we carried out a radial line scan of F-actin fluorescence intensity from the centrosome of resting Jurkat T cells. Based on its gradual decrease, we defined the centrosomal actin area as a circumference of 1 µm of radius around the MTOC **(** **Figure 5B** **)**. Immunofluorescence analysis also confirmed the presence of a centrosomal F-actin pool in Jurkat T cells in synapse with TCS-CD86 **(** **Figure 5C** **).** Control and SNX27-silenced cells presented a similar percentage of centrosomal F-actin MFI in basal conditions, which was equally reduced upon synapse formation with TCS-CD86 **(** **Figure 5D** **).** In agreement, conjugate formation induced a mild physical separation of the MTOC from the nucleus. The shorter distance between the edges of these two organelles was measured in three dimensions, and no significant differences in MTOC-nucleus detachment were found between control and SNX27-silenced cells **(** **Figures 5E, F** **).** These results demonstrate that centrosomal F-actin nucleation and the derived MTOC detachment from the nucleus are SNX27-independent.

### SNX27 Sustains Microtubule Organization at the IS

**Figure 6:**
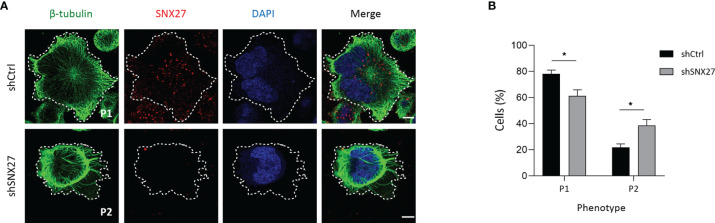
SNX27 silencing in Jurkat T cells affects microtubule reorganization at the IS. **(A)** Representative confocal images of control and SNX27-silenced Jurkat T cells stimulated on anti-CD3 and ICAM-1-coated coverslips, and immunostained for SNX27 (red) together with anti-β-tubulin (green). Images were post-treated by deconvolution. Representative maximum intensity projections of 4 consecutive sections (0.8 μm) at the cell-coverslip contact are shown (n = 3). Dashed white line indicates cell contour, which was identified by thresholding β-tubulin fluorescence intensity signal. Scale bar = 5 μm. **(B)** Quantification of microtubule network organization patterns at the IS, categorized by observation of unlabeled images by three independent investigators in two classes: pattern 1 (P1), radial microtubules anchoring at the periphery of the IS, or pattern 2 (P2), non-radial microtubules unable to reach the periphery of the IS. Data shown as mean ± SEM of three independent experiments run in duplicates (shCtrl = 131 cells, shSNX27 = 141 cells). Significance was determined by two-way ANOVA with Bonferroni correction (*p <0.05).

At the IS, microtubules growing from the MTOC radiate towards the periphery and anchor at the peripheral SMAC (pSMAC), characterized by dense LFA-1 clustering (57). In agreement, Jurkat T cells plated onto anti-CD3 and ICAM-1-coated coverslips showed a radially organized microtubule pattern, projecting to the periphery of the IS and with a visible translocated MTOC (defined as phenotype 1) **(** **Figure 6A**, top). SNX27-silenced Jurkat T cells more frequently displayed abnormal microtubule distribution that extended in filopodia-like shape unable to reach the periphery of the IS (defined as phenotype 2) (**Figure 6A**, bottom), as confirmed by image quantification (**Figure 6B**). Therefore, in addition to the mild defect in F-actin clearance at the IS and the failure to relocate the MTOC, SNX27-silenced Jurkat T cells present deficiencies in microtubule cytoskeleton organization at the IS. All in all, these results highlight a role for SNX27 in the control of cytoskeleton remodeling upon APC engagement.

### SNX27 Facilitates Signaling Microclusters Organization at the IS

T cell interaction with an APC triggers the activation and recruitment of signaling and adaptor molecules that assemble into microclusters. The formation of supramolecular activation clusters in T cell synapses was first shown in the late 90s by Monks and colleagues using fluorescence microscopy (58). Although TCR signaling takes place at the IS, not all the molecules involved in this process are found at the plasma membrane, and regulated vesicular trafficking is crucial for their assembly and organization at the cell-cell interface. Signaling molecules described to be localized at vesicular compartments include TCR, LAT, and Lck (4, 59–64). Remarkably, the traffic of these molecules to the IS is not determined by the same routes, resulting in the spatial organization of signaling microclusters with distinct compositions. Of note, some of the molecules involved in TCR signaling, such as Lck, ZAP70, LAT, SLP76, PLCγ1, and the scaffolding protein ADAP have been reported to play a key role in MTOC translocation to the IS (16, 65, 66). Moreover, radial microtubules at the IS and dynein were shown to contribute to the centripetal transport of TCR and SLP microclusters, as well as to the p-LAT pattern at the IS (67, 68).

**Figure 7:**
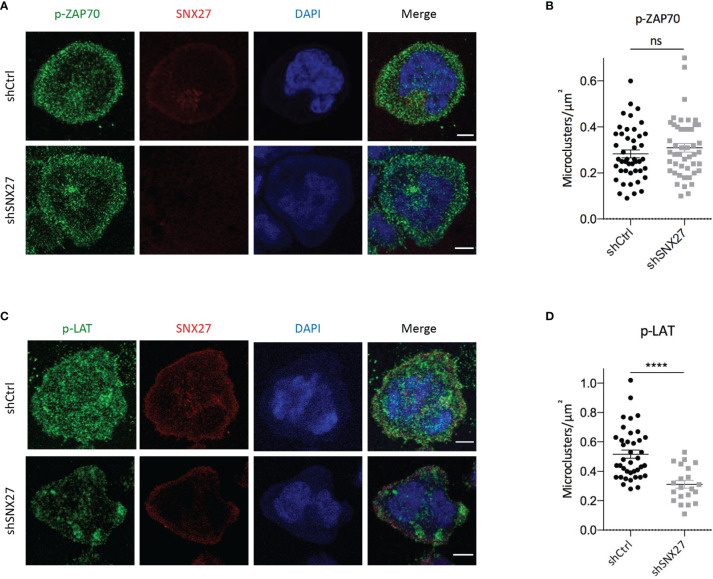
SNX27 silencing affects the pattern of p-LAT but not p-ZAP70 microclusters at the IS. **(A, C)** Representative confocal images of control and SNX27-silenced Jurkat T cells at the contact surface of anti-CD3 and ICAM-1-coated coverslips, immunostained for SNX27 (red), p-ZAP70 (Tyr 319) or p-LAT (Tyr 220) (green). DAPI was used to label the nuclei. Scale bar = 5 μm. **(B, D)** Quantification, at the cell-coverslip optical section, of p-LAT and p-ZAP70 microclusters density. Plot in **(B)** shows mean ± SEM of a representative experiment of two independent ones with similar results (shCtrl = 45 cells, shSNX27 = 47 cells). Plot in **(D)** shows mean ± SEM of a representative experiment out of three (shCtrl = 39 cells, shSNX27 = 21 cells). Significance was determined by unpaired *t* test (ns, not significant; ****p < 0.0001).

The finding that SNX27 silencing in Jurkat T cells affects cytoskeleton rearrangement, p150^Glued^, and MTOC synaptic recruitment prompted us to evaluate the relationship between SNX27 and microclusters organization at the IS. In order to induce the formation of signaling microclusters, we set control or SNX27-silenced Jurkat T cells to spread over stimulation surfaces coated with anti-CD3 and ICAM-1. We fixed cells after 10 minutes and analyzed the density of Tyr 319 phosphorylated ZAP70 (p-ZAP70) and Tyr 220 phosphorylated LAT (p-LAT) by immunofluorescence. While the analysis of p-ZAP70 microclusters did not show major differences between control and SNX27-silenced cells **(** **Figures 7A, B** **),** we observed that the density of p-LAT microclusters was decreased in SNX27-silenced cells **(** **Figures 7C, D** **).** This revealed that presence of SNX27 contributes to the correct arrangement of LAT signaling microclusters at the IS.

### SNX27 Limits Transcriptional Activation, IL-2 Production and Secretion

**Figure 8:**
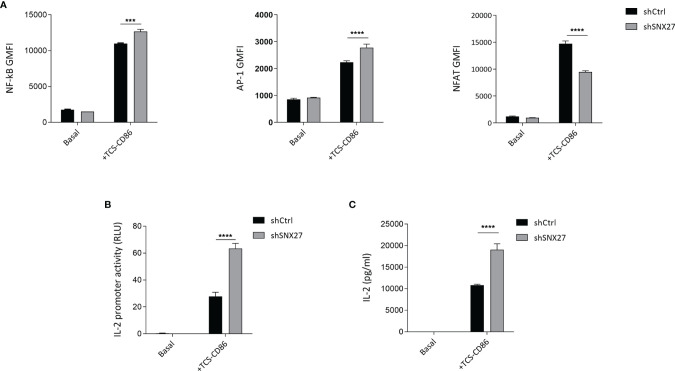
SNX27 silencing alters NF-κB, AP-1 and NFAT transcription factor activity and enhances IL-2 production and secretion. **(A)** Control or SNX27-silenced cells were stimulated with TCS-CD86 for 24 h NF-κB (left), AP-1 (middle) and NFAT (right) geometric mean fluorescence intensity (GMFI) activity were analyzed. **(B)** Luciferase activity of an IL-2 reporter construct was evaluated in control or SNX27-silenced Jurkat T cells stimulated with TCS-CD86 for 24 h Luciferase activity was corrected employing an internal renilla luciferase control. **(C)** IL-2 secretion was determined by ELISA in the supernatants of control or SNX27-silenced Jurkat T cells stimulated with TCS-CD86 for 24 h Data are shown as mean ± SEM of a representative experiment of 3 with similar results. Significance was determined by two-way ANOVA with Bonferroni correction (***p < 0.001, ****p < 0.0001).

Polarity regulators are crucial for T cell migration and cell remodeling upon encounter of an APC (69). Noteworthy, deficiency of some polarity proteins in T cells, such as ezrin, Dlg1 or adenomatous polyposis coli (Apc), lead to similar defects as the ones that we observe when silencing SNX27, such as impaired actin and microtubule reorganization at the IS or deficient MTOC translocation. These defects have been associated with altered T activation and effector function, such as hindered microclusters organization, decreased NFAT activation or IL-2 production (68, 70–72). In a previous study, we addressed in great detail the consequences of SNX27 silencing on T cell responses (33). Here we used the TPR Jurkat cell model engaged with TCS-CD86 to mimic the cell-cell contact between a T cell and an APC to further investigate the transcriptional control exerted by SNX27. TPR cells present response elements for NF-κB, NFAT, and AP-1 that drive the expression of the fluorescent proteins CFP, eGFP, and mCherry respectively, allowing assessment of these pathways by flow cytometry. (34). In agreement with previously reported data for DGKζ-silenced TPR cells (73), TCS-CD86 engagement of SNX27-silenced TPR cells promoted a more robust NF-κB and AP-1 induction but diminished that of NFAT compared to control cells (**Figure 8A** **)**. The inhibitory effect of SNX27 silencing on NFAT activation resembles that described when silencing other polarity regulators, suggesting common mechanisms leading to it, such as defective microtubule organization (70).

The increased NF-κB and AP-1 activity correlated with an augmented IL-2 transcription in SNX27-silenced Jurkat T cells, as demonstrated by measuring the transcriptional activation of a luciferase-coupled IL-2 promoter in Jurkat T cells stimulated with TCS-CD86 for 24 h (**Figure 8B**). Determination of IL-2 on the supernatant revealed that TCR co-stimulation by TCS-CD86 resulted in increased IL-2 secretion in SNX27-silenced cells compared to control ones (**Figure 8C**). These results are in agreement with the enhanced IL-2 production and secretion reported in SNX27-silenced Jurkat T cells upon stimulation with soluble anti-CD3/CD28 (33). Altogether these data indicate an important contribution of SNX27 in the correct activation of T cell programs.

### SNX27 Regulates Polarization of the Secretory Compartment

**Figure 9:**
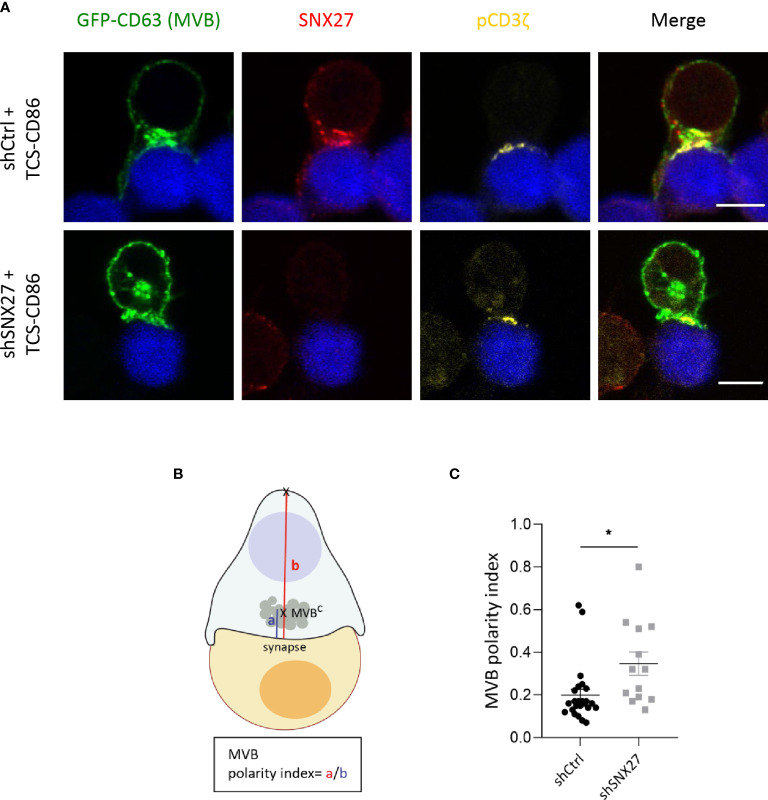
MBV recruitment to the IS is impaired in SNX27-silenced Jurkat T cells. **(A)** Representative confocal images of SNX27-silenced and control Jurkat T cells transfected with a GFP-CD63 construct (MVB), and incubated with TCS-CD86 (blue). Intracellular SNX27 (red) and phosphorylated CD3ζ (yellow) were stained as control of silencing and correct IS formation, respectively. Single medial optical sections from a representative experiment are shown (n = 2). Scale bar = 5 μm. **(B)** Graphical representation of polarity index calculation, computed as the ratio between the distance from the center of mass of the MVB (MVB^C^) to the IS (“a” distance) by the distance from the IS to the distal pole of the T cell (“b” distance). **(C)** MVB polarity index. Values are displayed as dot plots, with each dot representing an individual cell. Data shown as mean ± SEM of a representative experiment out of two (shCtrl = 24 cells, shSNX27 = 13 cells). Significance was determined by unpaired *t-test* (*p <0.05).

MTOC reorientation towards the IS facilitates polarized secretion of secretory granules and cytokines towards the cell-cell contact site (11, 74–76). It also mediates the polarization of MVB that fuse to the plasma membrane and release exosomes, favoring intercellular communication (12). Depletion of p150^Glued^ in Jurkat T cells impaired clustering of vesicles around the MTOC although it did not prevent MTOC translocation (77). To evaluate the contribution of SNX27 in MVB polarization to the IS, we transfected Jurkat T cells with a plasmid encoding for GFP-fused CD63 and followed its localization upon stimulation with costimulatory TCS-CD86 cells. The tetraspanin CD63 is enriched on the intraluminal vesicles of late endosomes/MVB, which are secreted as exosomes. Besides, it is also abundant on lysosomes and a small pool is present at the cell surface (78). In control Jurkat T cells stimulated with TCS-CD86 cells, SNX27 appeared to colocalize with GFP-CD63, which congregated near the IS **(** **Figure 9A**, up). In contrast, GFP-CD63 in SNX27-silenced was found at distal locations from the cell-cell contact region **(** **Figure 9A**, bottom). Staining of phosphorylated CD3ζ at the IS was used to confirm cell activation. Polarity index of the MVB compartment was computed as the ratio between the distance from the center of mass of the MVB (MVB^C^) to the IS (“a” distance) by the distance from the IS to the distal pole of the T cell (“b” distance) **(** **Figure 9B** **).** Calculation of MVB polarity indexes and percentage of conjugates with polarized MVB confirmed their impaired translocation in the absence of SNX27 **(** **Figure 9C** **).** Video-microscopy studies also corroborated the results observed in fixed conjugates (data not shown). This finding suggests that SNX27 is necessary for the efficient polarization of the exosome secretory machinery to the IS.

## Discussion

The polarization of the MTOC and the secretory machinery to the IS represent two mechanisms indispensable for correct T cell functions. SNX27 best characterized role is that of facilitating PDZ-mediated rapid recycling of its transmembrane cargoes from endosomes to the plasma membrane, avoiding their lysosomal degradation (8, 25, 28). Here we add a novel and important function for SNX27 by showing that it acts as a hub for adequate MTOC repositioning and polarization of secretory compartments in T lymphocytes upon APC engagement.

Despite being widely recognized as a critical event in lymphocyte function, the mechanisms driving MTOC translocation in T cells are not completely understood. Here we describe multiple, complementary mechanisms by which SNX27 translocation to the IS may be involved in this process. Firstly, we demonstrate that SNX27 is critical for the regulation of synaptic DAG accumulation, one of the reported triggers required for MTOC polarization. Secondly, we describe a mild defect in F-actin depletion across the synapse in SNX27-silenced cells. Thirdly, we report how SNX27 deficiency leads to randomly organized microtubules, unable to connect the MTOC to the cortex at the pSMAC.

Perturbation of the DAG gradient established during IS formation has been previously linked with impaired MTOC polarization (14–16). Reinforcing this notion, impaired DAG accumulation in SNX27-deficient cells correlates with defective MTOC orientation. The observed defects in DAG content at the IS following SNX27 silencing could be, at least partially, the consequence of abnormal localization of DGKζ. Several studies have reported substantial defects in the organization of the IS consequent to DGK deficiency. For instance, activated CD4^+^ mouse T cells treated with DGK inhibitors or deficient for DGKα presented impaired DAG accumulation and MTOC recruitment to the IS (14, 16). Besides, DGKζ-deficient CTLs were unable to dock the MTOC to the IS (46), and DGKζ-deficient B cells presented impaired actin remodelling, force generation and MTOC translocation at the IS (79). Our studies show that not only the expression but also the correct spatial distribution of DGKζ are important for the regulation of DAG content in basal conditions and for the generation of DAG gradients upon antigen recognition. SNX27-dependent PDZ interaction allows retrograde DGKζ traffic to internal regions, that in turn facilitate its polarized recruitment to the IS. The high DAG amount in internal membranes derives to great extent from PA hydrolysis by PA phosphatases (39, 80). The failure of DGKζ to reach internal compartments as the results of SNX27 silencing could thus prevent an adequate supply of PA and ultimately lead to the shutdown of PA-dependent DAG generation.

The defect on MTOC translocation in the absence of SNX27 appears much stronger than the partial defect described in DGKζ-deficient CTLs and B cells, suggesting additional mechanisms governed by SNX27. As we show, SNX27-silenced cells display a mild defect in F-actin depletion across the IS that could contribute to the failure in MTOC polarization. Although studies in B cells and Jurkat T cells described that MTOC reorientation is independent of F-actin reorganization at the IS (74, 81, 82), other numerous investigations reported that it does play an important role in this mechanism (49–53). Recent studies have proposed that depletion of centrosomal F-actin by WASH-dependent mechanisms is required for MTOC translocation in Jurkat T cells upon IS triggering (54). However, in our hands, SNX27 silencing did not affect centrosomal F-actin reduction nor MTOC detachment from the nucleus upon engagement with TCS-CD86. Therefore, WASH functions at the IS and centrosomal area might be independent on SNX27 interaction.

Defects in microtubule reorganization during IS formation following SNX27 silencing correlates well with impaired p150^Glued^ translocation to the cell-cell contact region following SNX27 silencing. Although the contribution of microtubule dynamics or stabilization to MTOC reorientation remains unresolved, inhibition of microtubule polymerization in human primary CD4^+^ T cells and Jurkat T cells has been described to block MTOC translocation towards the APC (83, 84). Dynactin directly binds microtubules and cytoplasmic dynein, which stabilizes the association between dynein and its cargoes and facilitates their retrograde transport along the microtubule cytoskeleton (85–87; 88). In T cells, this complex is recruited to the IS upon TCR activation and has been described to be of great relevance for MTOC polarization (16, 65, 84, 89). Disruption of the dynein-dynactin complex at the IS by overexpression of p50-dynamitin-GFP impairs MTOC translocation. to the contact area between Jurkat and SEE-pulsed Raji cells (89). Localization of p150^Glued^ at microtubules and the MTOC (77) correlates with our studies and identify SNX27 as essential for the correct localization of this protein. SNX27-retromer interaction with p150^Glued^ would facilitate its transport to the IS, favoring the anchoring of this complex to the microtubules minus ends. In turn, this molecular motor would exert a pulling force on microtubules, dragging the attached MTOC to the IS. Further research on the relationship between SNX27 and p150^Glued^ will likely shed light on the exact mechanism linking SNX27 to the control of MTOC polarization.

SNX27 contribution to microtubule rearrangement has a direct impact in the correct organization of p-LAT microclusters. Phosphorylated LAT constitutes a docking site for multiple signaling and adaptor proteins such as the adaptor SLP76 and PLCγ1 (90; 91). PLCγ1 is responsible for initial generation of DAG at the cell-cell contact zone. Therefore, the defects observed in the organization of p-LAT microclusters in SNX27-silenced cells could translate into inappropriate PLCγ1 recruitment and activity. This would contribute to the impaired formation of a synaptic DAG gradient observed in these cells. These predicted outcomes are supported by reported data describing that defects in microtubule polymerization at the IS do not affect ZAP70 activation, but are associated with defective LAT activation and synaptic accumulation, as well as decreased PLCγ1 phosphorylation (92). Interestingly, silencing ezrin or its partner, the polarity regulator Dlg1, hinders microtubule anchoring to the cortical actin cytoskeleton, and its consequences are very much reminiscent of the alterations described here: defective MTOC polarization, microtubule network organization at the IS and p-LAT microcluster patterns. In addition, it is accompanied by defects in microcluster centripetal transport (68). Moreover, defects in the polarity regulator Apc, a partner of Dlg1, induces similar defects on microtubule organization patterns, actin clearance and cytotoxic granule localization and fusion at the IS (70, 71). Both Dlg1 and Apc are components of cell polarity complexes interacting through PDZ/PDZbm domains (93). SNX27 in T cells could be contributing to regulate the coordinated action of cytoskeleton and vesicular traffic at the IS through the same mechanism, as it has been reported to interact with a partner of these cell polarity complexes termed β-PIX (44).

DGKζ-silenced Jurkat T cells enhanced activation of PKCθ downstream TCR/CD28 stimulation, which has been directly related to increased NF-κB-mediated transcription (94). Luciferase studies in SNX27-silenced Jurkat T cells confirmed an increased NF-κB activation that mirrored the one observed upon DGKζ silencing. Dual silencing of SNX27 and DGKζ had no additive effect, suggesting that SNX27 interaction with DGKζ sustained its function as a negative regulator of DAG metabolism (44). This is in accordance with our observation that SNX27 facilitates the control exterted by DGKζ on the correct formation of a synaptic DAG-gradient. In the current study, we further investigated the transcriptional regulation by SNX27 in IS-forming TPR cells. SNX27-silenced T cells stimulated with TCS-CD86 display increased AP-1 and NF-κB-dependent transcription, as well as augmented IL-2 secretion compared to control cells, an effect similar to that described for DGKζ-silenced TPR cells (73). The use of this model confirms previous luciferase studies in CD3/CD28-stimulated Jurkat T cells and demonstrates the strict regulation of NF-κB transcription by SNX27. Although IL-2 has been reported to focus at the IS closely associated with the MTOC, our data indicate that MTOC polarization is not required for the secretion of this cytokine. This finding is supported by previous studies showing enhanced IL-2 and IFN-γ secretion by T cells with impaired MTOC synaptic translocation (74, 95).

SNX27-silenced TPR cells display decreased NFAT-dependent activation upon TCS-CD86 engagement. Of interest, silencing of the polarity regulator Apc leads to deficiencies in microtubule organization in T cells, which in turn impair NFAT nuclear translocation and its mediated transcription (70, 71). In addition, other polarity proteins such as Dlg1 and ezrin control NFAT activation by alternative p38 activation (68, 72). In this study we show that SNX27 is involved in microtubule remodeling and synaptic organization of p-LAT microclusters. Therefore, we suggest that SNX27 silencing could be triggering a decreased NFAT activation through similar mechanisms as the described for the aforementioned polarity regulators. Further research will help to shed light to this issue.

In summary, our study reveals several meaningful details about the function of SNX27 to maintain a polarized intracellular organization, constituting an important regulator of cytoskeletal organization and T cell activation during IS formation. These results could also be of relevance in the analogous neuronal synapse. It is remarkable that some of the interactors identified in the Jurkat proteomic study are proteins with critical functions in centrosome orientation, whose defects are related to human and mice microcephaly due to impaired centrosomal localization during neurogenesis. This is the case of CENPJ where mutations are associated with microcephaly and Seckel syndrome (96, 97); citron kinase whose loss of activity leads to human microcephaly (98), and a malformative syndrome in mice (99); as well as β-PIX and GIT1 where mutations have been linked to intellectual disability and microcephaly (100–104). Additional research on SNX27-regulated functions will likely shed additional light on immune and neuronal synapse function and dysfunction.

## Data Availability Statement

The raw data supporting the conclusions of this article will be made available by the authors, without undue reservation.

## Author Contributions

NG-M: experimental procedures, data analysis, preparation of figures, original draft preparation. CR-R: experimental procedures, preparation of figures, original draft preparation. AA: conceptualization and coordination of the research, review. IM: conceptualization and coordination of the research, original draft preparation and review. All authors read and agreed to the published version of the manuscript.

## Funding

NG-M received funding from the European Union Horizon 2020 research and innovation program under the Marie Sklodowska-Curie grant agreement 713673 and “La Caixa” Foundation (ID 100010434), with the fellowship code being LCF/BQ/DI17/11620027, as well as an EMBO short-term fellowship. CR-R held a predoctoral fellow of the Álvaro Entrecanales and Jérôme Lejeune Foundations. Research in IM’s lab is funded by grants from the Spanish Association Against Cancer (AECC, CICPF18), Aplastic Anemia and MDS International Foundation (AAMDSIF, OPE01644), Spanish Ministry of Science and Innovation (PID2019-108357RB-100/AEI/10.13039/501100011033), and the Madrid regional government (IMMUNOTHERCAM Consortium S2010/BMD-2326) to IM. Research in AA’s lab is funded by the French Ligue Nationale Contre le Cancer, Equipe Labellisée 2018).

## Conflict of Interest

The authors declare that the research was conducted in the absence of any commercial or financial relationships that could be construed as a potential conflict of interest.

## Publisher’s Note

All claims expressed in this article are solely those of the authors and do not necessarily represent those of their affiliated organizations, or those of the publisher, the editors and the reviewers. Any product that may be evaluated in this article, or claim that may be made by its manufacturer, is not guaranteed or endorsed by the publisher.

## Supplementary Material

Supplementary Figure 1 DAG generation at the plasma membrane of the IS precedes the full translocation of SNX27 and DAG-enriched compartments. Representative maximum intensity projections of control Jurkat T cells transfected with the C1 domain of PKCθ fused to GFP construct (GFP-C1bPKCθ, green) after incubation with TCS-CD86 (blue). Cells were immunostained for SNX27 (red). Arrowhead points DAG in intracellular compartments, while arrows show DAG generation at the plasma membrane of the IS. Scale bar = 5 μm.
