# Supplementary figures and images for: Sorting Nexin 27 Enables MTOC and Secretory Machinery Translocation to the Immune Synapse
Source: Front Immunol. 2022 Jan 12;12:814570. doi: 10.3389/fimmu.2021.814570 (PMC8790036; doi:10.3389/fimmu.2021.814570)

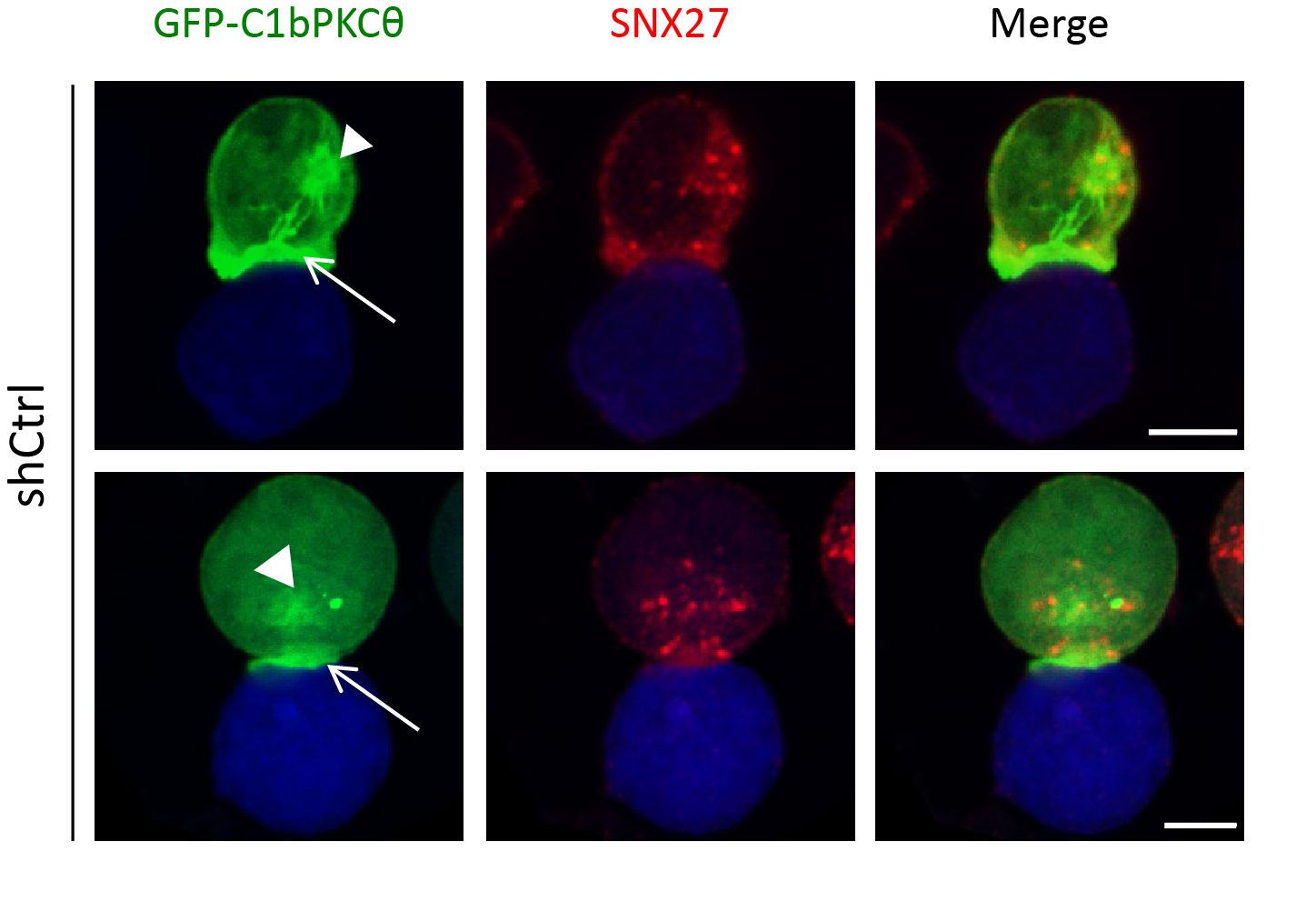

Supplement: Supplementary Figure 1 — DAG generation at the plasma membrane of the IS precedes the full translocation of SNX27 and DAG-enriched compartments. Representative maximum intensity projections of control Jurkat T cells transfected with the C1 domain of PKCθ fused to GFP construct (GFP-C1bPKCθ, green) after incubation with TCS-CD86 (blue). Cells were immunostained for SNX27 (red). Arrowhead points DAG in intracellular compartments, while arrows show DAG generation at the plasma membrane of the IS. Scale bar = 5 μm. [file Image_1.tiff]
